# Epidemiological and aetiological characteristics of hand, foot, and mouth disease in Shijiazhuang City, Hebei province, China, 2009-2012

**DOI:** 10.1371/journal.pone.0176604

**Published:** 2017-05-09

**Authors:** Huifang Tian, Yong Zhang, Yan Shi, Xiujuan Li, Qiang Sun, Li Liu, Dong Zhao, Baohong Xu

**Affiliations:** 1 Shijiazhuang Center for Disease Control and Prevention, Shijiazhuang City, Hebei Province, People’s Republic of China; 2 WHO WPRO Regional Polio Reference Laboratory and Key Laboratory for Medical Virology, National Health and Family Planning Commission of China; National Institute for Viral Disease Control and Prevention, Chinese Center for Disease Control and Prevention, Beijing, People’s Republic of China; Kliniken der Stadt Köln gGmbH, GERMANY

## Abstract

Large outbreaks of hand, foot, and mouth disease (HFMD) have repeatedly occurred in mainland of China since 2007. In this study, we investigated the epidemiological and aetiological characteristics of HFMD in Shijiazhuang City, one of the biggest northern cities of China. A total of 57,173 clinical HFMD cases, including 911 severe and 32 fatal cases, were reported in Shijiazhuang City during 2009–2012. The disease incidence peaked during March–July, with a small increase in the number of cases observed in November of each year. Seventeen potential HFMD-causing enterovirus serotypes were detected, with the most frequent serotypes being EV-A71 and CV-A16. CV-A10 was also a frequently detected causative serotype, and was associated with the second largest number of severe HFMD cases, following EV-A71. Phylogenetic analysis revealed that all EV-A71, CV-A16 and CV-A10 strains from Shijiazhuang City had co-evolved and co-circulated with those from other Chinese provinces. Our findings underscore the need for enhanced surveillance and molecular detection for HFMD, and suggest that EV-A71 vaccination may be an effective intervention strategy for HFMD prevention and vaccines against CV-A10 and CV-A16 are also urgently needed.

## Introduction

Hand, foot, and mouth disease (HFMD) is a common infectious disease in young children, particularly those aged <5 years, and the disease is more prevalent during the warm season (May to July)[1–4]. HFMD is usually caused by human enteroviruses (EV) that are members of the genus *Enterovirus* within the family *Picornaviridae*, order *Picornavirales*, consisting of four species: *EV-A*, *EV-B*, *EV-C*, and *EV-D*. Till date, EV comprises more than 100 serotypes that are the most common pathogens infecting humans, especially children, worldwide.

Most EV infections are asymptomatic or present with benign symptoms; are characterised by a brief febrile illness, typical vesicular rashes (on the palms, soles or buttocks), and oropharyngeal ulcers; and can usually resolve spontaneously. Occasionally, however, they may also lead to serious neurological complications such as acute flaccid paralysis, encephalitis, myocarditis, and encephalomyelitis[5–7]. HFMD can be transmitted by direct person-to-person contact through nasal discharge, saliva, faeces, and fluid from blisters of infected persons. Additionally, it can spread through EV-contaminated water and food[8, 9].

As is well established, *EV-A* and *EV-B* account for most of the HFMD cases. The most common aetiological agents of HFMD are two members of the *EV-A* species—enterovirus A71 (EV-A71) and coxsackievirus A16 (CV-A16), with varying incidence rates attributed to each[8, 10–13]. The first CV-A16 infection was reported in Canada, and it is one of the most widely prevalent EV serotypes in the world. The first reported EV-A71 infection occurred in the United States, and the serotype has been a recurrent feature in the Asia-Pacific region since the first reported outbreak in Sarawak, Malaysia in 1997[14–17]. Besides these two serotypes, other *EV-A* and *EV-B* serotypes may also co-circulate and account for a sizeable proportion of the pathogen spectrum of HFMD; the clinical features of the resultant infections are usually indistinguishable from those caused by the two predominant EV serotypes [18–21]. Among these other serotypes, CV-A6 is especially important as it is known to have circulated in mainland of China and was the predominant HFMD-causing EV in 2013 and 2015[22–25].

Large outbreaks of HFMD have repeatedly occurred in mainland of China since 2007, and the disease has become a serious public health concern[9, 26]. Over the past few years, some studies on the epidemiological characteristics of HFMD have been carried out in mainland of China, especially in central and southern China[27–30]. Shijiazhuang city is the capital of Hebei province, located in the north of China, and is one of the biggest Chinese cities. During 2009–2012, 51,573 HFMD cases were reported in Shijiazhuang City through the National Notifiable Disease Reporting System (NNDRS). In this study, we aimed to characterise the epidemiology and aetiology of HFMD in the Shijiazhuang City and to identify the spectrum of HFMD-causing pathogens. Besides EV-A71 and CV-A16, CV-A10 could also be one of the main pathogens that caused the HFMD outbreaks during 2009–2012. Our results indicate the need for enhanced EV surveillance to better understand the aetiology of HFMD, and to meet the emergency needs in case of outbreaks triggered by these EV serotypes.

## Results

### Demographic, temporal and geographical distribution characteristics of HFMD epidemiology in the Shijiazhuang City

**Fig 1:**
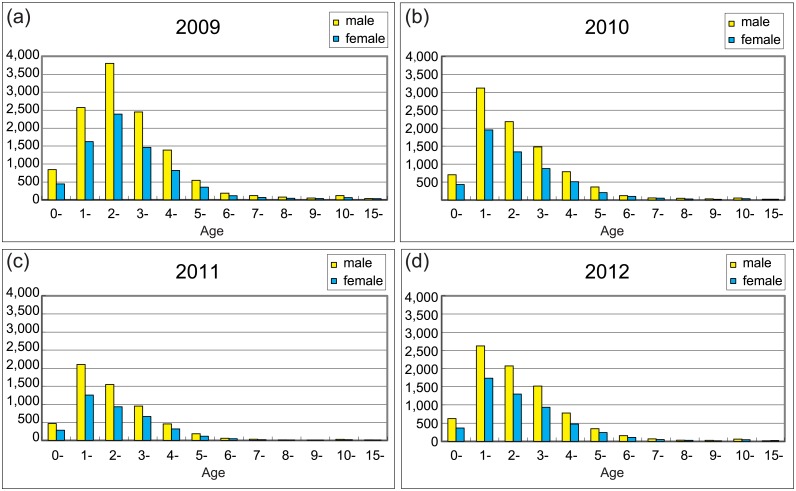
Number of HFMD cases in Shijiazhuang city in 2009 (a), 2010 (b), 2011 (c), and 2012 (d), stratified by age and gender.

According to the case definition criteria set forth in the national guidelines for control and prevention for HFMD (issued by Ministry of Health in China in 2009), a total of 57,173 clinical HFMD cases were reported to the (national notifiable disease report system, NNDRS) at the Shijiazhuang Center for Diseases Control and Prevention (Shijiazhuang CDC) during 2009–2012, including 911 severe and 32 fatal cases (Table 1). The average annual HFMD incidence rate was 142.97 per 100,000 population (range, 96.24–201.42 per 100,000 population). The lowest number of annual HFMD cases was observed in 2011 (9,550), and the highest number was reported in 2009 (19,446), forming the peak and trough in the HFMD incidence. The majority (79.93%) of HFMD cases occurred in scattered children, while a small proportion of occurred in kindergarten (17.36%). Of the HFMD cases reported during 2009–2012, 35,212 were male patients and 21,961 were female patients, with an average annual male-to-female ratio of 1.61:1 (range, 1.56:1–1.63:1), suggesting that male children were relatively more susceptible to HFMD virus infection than female children. Of all children, 90.85% (51,942) were younger than five years old, accounting for 90.45% (17,589), 91.97% (13,361), 93.98% (8,975), and 91.09% (12,434) of the reported cases in 2009, 2010, 2011, and 2012, respectively (Fig 1).

**Table 1:** The epidemiological features of HFMD cases reported in 2009–2012 from Shijiazhuang city, Hebei province, China, via the National Notifiable Disease Reporting System.

Year	Number of patients	Sex ratio (male: female)	Reported incidence rate (per 100,000)	Number of patients under 5 years old (%)	Number of severe cases	Number of fatal cases	Scattered children cases (%)	Kindergarten hosting cases (%)	Other facilities cases (%)
2009	19,446	1.63	201.42	17,589 (90.45)	133	0	16,606 (79.85)	3,407 (17.52)	511 (2.63)
2010	14,527	1.61	140.06	13,361 (91.97)	537	28	11,031 (82.32)	2,204 (15.17)	365 (2.51)
2011	9,550	1.59	96.24	8,975 (93.98)	121	2	7,671 (81.74)	1,533 (16.05)	211 (2.21)
2012	13,650	1.56	134.14	12,434 (91.09)	120	2	10,391 (76.24)	2,778 (20.35)	465 (3.41)
Total	57,173	1.61	142.97	51,942 (90.85)	911	32	45,699 (79.93)	9,922 (17.36)	1,552 (2.71)

**Fig 2:**
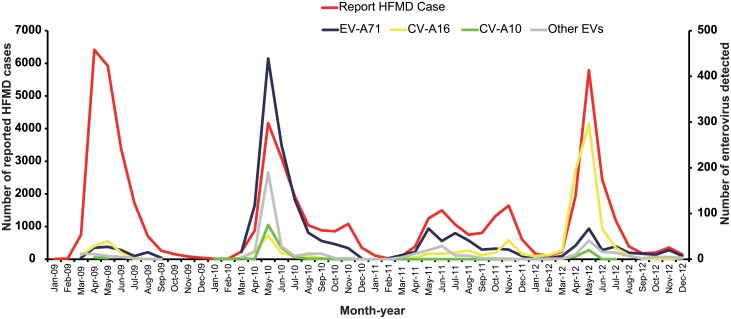
Monthly distribution of HFMD cases and enteroviruses detected in Shijiazhuang City, Hebei province of China, 2009–2012. The number of HFMD cases had apparent seasonal distribution with the peaks in April (year 2009), May (years 2010 and 2012) and June (year 2011), respectively.

Although HFMD cases occurred throughout the year, a seasonal increase in HFMD cases was observed from March to July each year, accounting for 76.15% of all annual cases. This monthly peak was observed in April in 2009, May in 2010 and 2012, and June in the year 2011, respectively (Fig 2). Notably, there was a small increase in the number of HFMD cases in November during the 2010–2012 period. This is a common phenomenon in the southern provinces of China, but rare in the Northern provinces[8].

**Fig 3:**
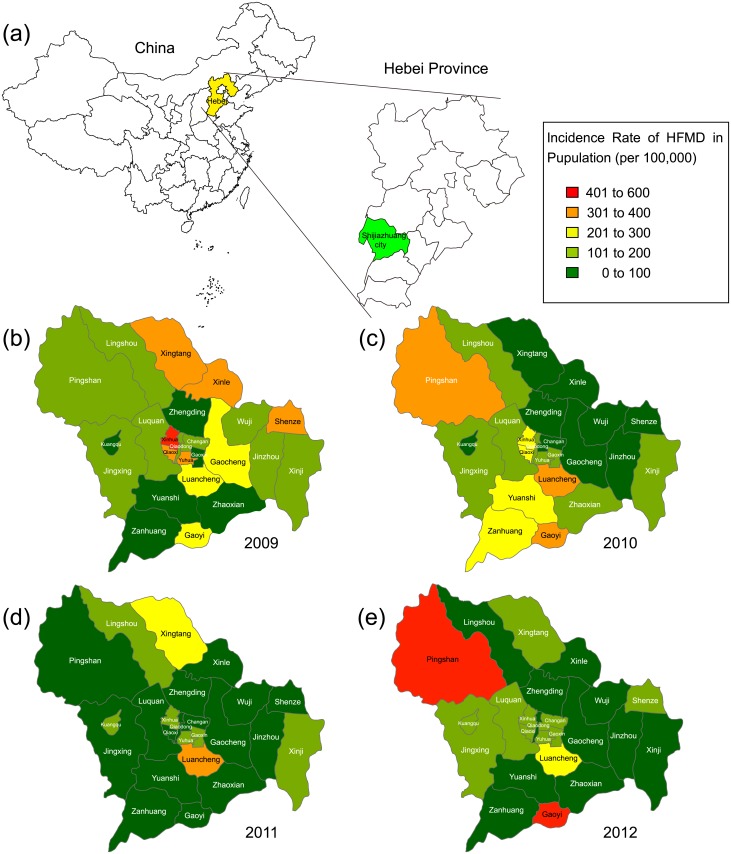
The geographic distribution of HFMD cases in Shijiazhuang city, Hebei province of China, 2009–2012. The epidemic covered all 24 districts of Shijiazhuang City, and annual HFMD incidence rates at the district/county level were indicated in different colours. The maps were generated with MapInfo Pro software (version 16.0, http://www.pitneybowes.com/us/location-intelligence/geographic-information-systems/mapinfo-pro.html).

The HFMD epidemic affected all 24 districts/counties of the Shijiazhuang City; however, clear differences in the annual HFMD incidence among the different districts/counties were observed. Moreover, the disease prevalence demonstrated a tendency to spread from the central urban area to the peripheral suburban areas during 2009–2012, which may reflect a pattern of transmission from a densely populated area to the surrounding areas (Fig 3). During the peak of HFMD epidemic in 2009, the incidence rate in six districts/counties exceeded 300 per 100,000 population, with the highest rate observed in the central urban Xinhua district (409.14 per 100,000 population). From 2010 to 2011, the incidence was relatively low with most districts/counties exhibiting incidence rates lower than 200 per 100,000 population. A second peak of the epidemic occurred in 2012, during which, high incidence rates were observed in the peripheral suburban areas of the Shijiazhuang City. The highest incidence rate during this epidemic peak was noted in the northern (Pingshan County, 442.53 per 100,000 population) and southern suburban areas (Gaoyi County, 526.10 per 100,000 population).

### Pathogenic spectrum and aetiological characterization of HFMD in Shijiazhuang City

Among the 57,173 clinical HFMD cases reported between 2009 and 2012, 3,050 (5.3%) cases, including 607 severe and 15 fatal cases, were laboratory-confirmed. The most frequently present EV serotypes were EV-A71 (52.8%) and CV-A16 (31.2%); co-infection with these viruses was found in very few cases (0.4%) (Table 2). However, the circulating trends of the two viruses showed a temporal shift during the epidemic. The prevalence of EV-A71 increased to reach a peak in 2010 (accounting for 74.0% of all HFMD-causing pathogens compared to 37.4% in 2009) and declined thereafter during the 2011–2012 period (57.3% in 2011 and 22.8% in 2012). The epidemic pattern of CV-A16 was completely different from that observed for the EV-A71. The CV-A16 demonstrated two epidemic peaks in 2009 and 2012 (accounting for 46.2% and 65.7% of all HFMD-causing pathogens, respectively), while its prevalence was relatively less in 2010 and 2011 (7.1% and 26.3% of all pathogens, respectively).

**Table 2:** Aetiological composition of the laboratory-confirmed cases in Shijiazhuang City, Hebei province, China, 2009–2012. The analysis was stratified by years, clinical manifestations and seasons.

Characteristics	Laboratory-confirmed cases	Aetiological composition				
		EV-A71 (%)	CV-A16 (%)	EV-A71 and CV-A16 coinfection (%)	CV-A10 (%)	Other EVs (%)
Total	3,050	1610 (52.8)	951 (31.2)	13 (0.4)	142 (4.7)	334 (10.9)
Year of isolation^▲^						
2009	182	68 (37.4)	84 (46.1)	0 (0.0)	7 (3.8)	23 (12.7)
2010	1,382	1,022 (74.0)	98 (7.1)	8 (0.5)	108 (7.8)	146 (10.6)
2011	525	301 (57.3)	138 (26.3)	0 (0.0)	0 (0.0)	86 (16.4)
2012	961	219 (22.8)	631 (65.7)	5 (0.5)	27 (2.8)	79 (8.2)
Clinical manifestation^★^						
Fatal	15	13 (86.7)	0 (0.0)	0 (0.0)	0 (0.0)	2 (13.3)
Severe	607	511 (84.2)	14 (2.3)	4 (0.6)	29 (4.8)	49 (8.1)
Mild	2,428	1,086 (44.7)	937 (38.6)	9 (0.4)	113 (4.7)	283 (11.7)
Seasonal distribution^◆^						
Winter (Dec to Feb)	50	18 (36.0)	27 (54.0)	0 (0.0)	0 (0.0)	5 (10.0)
Spring (Mar to May)	1,728	745 (43.1)	667 (38.6)	8 (0.5)	110 (6.4)	308 (17.8)
Summer (Jun to Aug)	964	641 (66.5)	188 (19.5)	5 (0.5)	31 (3.2)	130 (13.5)
Autumn (Sep to Nov)	308	206 (66.9)	69 (22.4)	0 (0.0)	1 (0.3)	33 (10.7)

^▲^Pearson’s Chi-square test: χ^2^ = 956.063, *P*<0.001.

^★^Pearson’s Chi-square test: χ^2^ = 361.024, *P*<0.001.

^◆^Pearson’s Chi-square test: χ^2^ = 183.566, *P*<0.001.

The 2010 HFMD epidemic peak is mainly caused by EV-A71 and the 2012 HFMD epidemic peak is mainly caused by CV-A16; while the 2011 HFMD epidemic peaks are a bit complicated, EV-A71 related to the summer peak and CV-A16 related to the autumn peak. However, there is no time delay between the time series of HFMD cases and pathogens (Fig 2).

EV-A71 was more frequently identified in fatal (86.7%), and severe cases (84.2%) than in mild cases (44.7%), while CV-A16 was more frequently identified in mild cases (38.6%); no CV-A16-associated fatal cases were identified. Statistically, significant differences were observed between the identified viruses and their associated clinical manifestations (Table 2).

**Fig 4:**
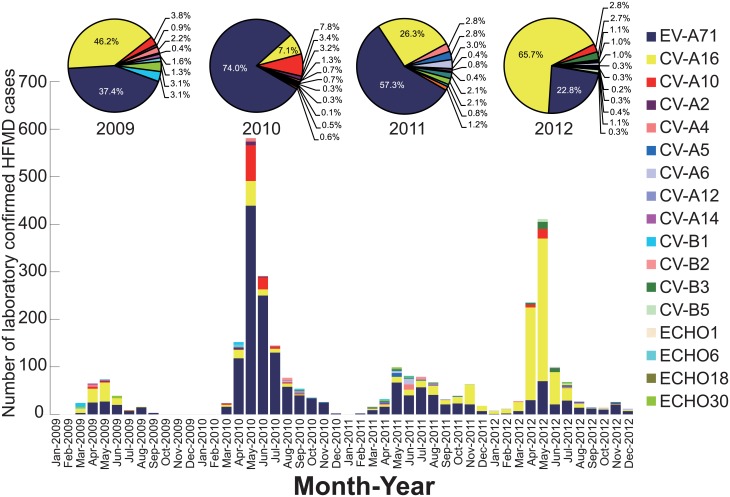
Monthly distributions and constituent ratio of enterovirus serotypes associated with laboratory-confirmed HFMD cases in Shijiazhuang City, Hebei province of China, 2009–2012.

The month-wise distribution of different EV serotypes is illustrated in Fig 4. The dominant EV serotypes, such as EV-A71 and CV-A16, were isolated during the yearly HFMD-seasonal peak (March–August) when most HFMD cases were reported. It should be noted that the pathogen spectrum can sometimes change, for example, the proportion of CV-A10 was higher than CV-A16, becoming the second highest detected HFMD-causing virus in 2010 (Fig 4).

In addition to the EV-A71 (1610 cases, 52.8%) and CV-A16 (951 cases, 31.2%) serotypes, CV-A10 (142 cases) was also the frequently detected serotype and caused HFMD outbreaks during 2009–2012, accounting for 4.7% of all laboratory-confirmed cases. Importantly CV-A10 was the causative pathogen in 29 (4.8%) severe cases and resulted in the second-highest number of severe cases, following the EV-A71 serotype. Similar to EV-A71 and CV-A16, the CV-A10 prevalence was also observed in the warm season (April to June). CV-A10 caused two HFMD outbreaks in 2010 and 2012, respectively (Fig 4); the 2010 CV-A10 epidemic (108 laboratory-confirmed cases) was bigger with a peak reported in the months of May and June.

**Fig 5:**
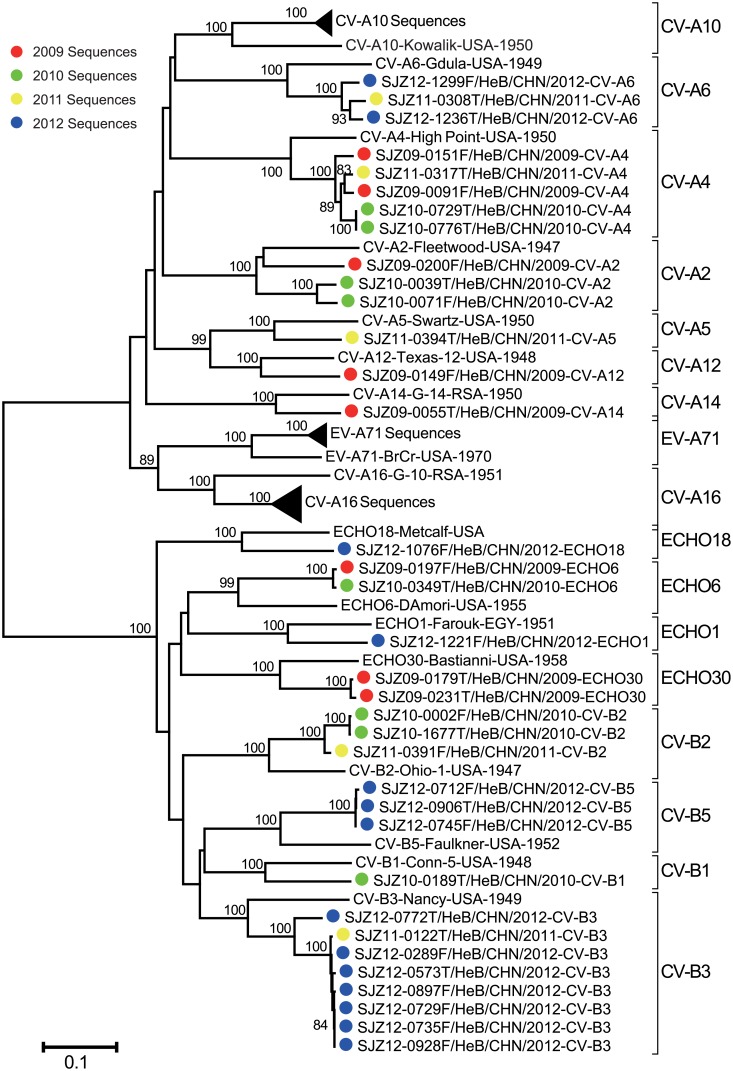
Phylogenetic tree based on the entire *VP1* sequences of the enteroviruses from HFMD cases. EV-A71, CV-A16, and CV-A10 sequences were omitted in the tree due to large number of the sequences, and sequences of this study were indicated by different colour solid circle in 2009 (red), 2010 (green), 2011 (yellow), and 2012 (blue).

Molecular typing identified 35 HFMD-associated non-EV-A71-, non-CV-A16-, non-CV-A10 EVs of 14 serotypes: CV-A2 (3 cases); CV-A4 (5 cases); CV-A5 (1 case); CV-A6 (3 cases); CV-A12 (1 case); CV-A14 (1 case); CV-B1 (1 case); CV-B2 (3 cases); CV-B3 (8 cases); CV-B5 (3 cases); ECHO1 (1 case); ECHO6 (2 cases); ECHO18 (1 case) and ECHO30 (2 cases), with different annual detection proportions. The molecular typing results were confirmed using a phylogenetic analysis (Fig 5).

### Phylogenetic analysis of the entire VP1 region of EV-A71, CV-A16, and CV-A10 isolates

To genetically characterise the three most frequently identified pathogens (EV-A71, CV-A16, and CV-A10) that caused HFMD in the Shijiazhuang City during 2009–2012, the entire *VP1* regions of 13 EV-A71, 19 CV-A16 and 14 CV-A10 isolates were randomly selected to sequencing. The *VP1* sequences of EV-A71 isolates in this study were closely related to each other, sharing 94.16–99.66% nucleotide similarity and 98.65–100% amino acid similarity. The *VP1* sequences of the CV-A16 and CV-A10 isolates shared 90.46–100% and 94.74–100% nucleotide similarity, respectively, corresponding to 98.99–100% and 98.32–100% amino acid similarity, respectively.

**Fig 6:**
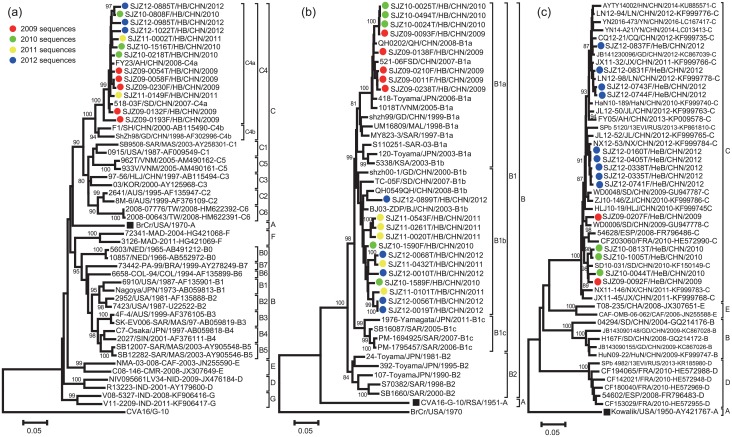
Phylogenetic tree based on the entire *VP1* coding region depicting the clustering of (a) EV-A71, (b) CV-A16, and (c) CV-A10 isolated in HFMD cases in Shijiazhuang City, 2009–2012. The viruses isolated from this study are marked different colour solid circle in 2009 (red), 2010 (green), 2011 (yellow), and 2012 (blue). In addition, the prototype virus is marked in black solid square.

The above-mentioned nucleotide and amino acid sequence similarities were also reflected in the phylogenetic tree-based analysis of the entire *VP1* sequences. All Shijiazhuang EV-A71 isolates, together with other EV-A71 strains isolated from HFMD cases in mainland of China, clustered to genotype C4, the C4a evolutionary branch (Fig 6a). Similarly, all Shijiazhuang and mainland of China CV-A16 isolates, clustered to genotype B1, both evolutionary B1a and B1b evolutionary branches (Fig 6b). Furthermore, all Shijiazhuang CV-A10 isolates, together with most CV-A10 strains isolated from mainland of China, clustered to genotype C, with only a few Chinese CV-A10 strains isolated between 2004–2009 clustering to genotype B (Fig 6c). Of note, all EV-A71, CV-A16 and CV-A10 strains in this study were observed to cluster with the corresponding EV serotypes isolated from other provinces of mainland of China, suggesting their co-evolution and co-circulation with viruses from other Chinese provinces.

## Discussion

During the 2009–2012 epidemic, a total of 51,573 HFMD cases were reported in the Shijiazhuang City, Hebei Province, China. The epidemiological characteristics of the Shijiazhuang City HFMD epidemic demonstrated many similarities with previous large outbreaks in the Asia-Pacific region[31, 32]. Seasonal models, predicting the outbreaks of HFMD cases during warmer climate conditions, were used to explain the annual fluctuations in HFMD prevalence in Tokyo, Japan[33]. Our findings are consistent with this view as the number of HFMD cases increased each year during April–May every year, to accompany the advent of warmer weather, suggesting that higher temperature increases the likelihood of HFMD spread and transmission. We also observed a small increase in the number of HFMD cases in November of every year, a phenomenon commonly reported in the southern provinces[8, 29, 34] but rare in the northern provinces of China due to the different climate conditions (temperature, air pressure and the amount of sunshine, etc.) prevalent in the month of November. The periodicity of the two epidemic peaks might be complicated by interference among the different causative EV serotypes; however, this has not been understood fully till now and deserves further investigation.

Moreover, consistent with the findings from other countries and regions[35–37], 99.7% of the HFMD cases in the Shijiazhuang City (56,995) were <10-year old children, and 91.1% of all reported HFMD cases were <4-year old children. Young children (<4 years old) are thus most susceptible to HFMD infection and should be the target population for HFMD prevention. In mainland of China, the 0–4 years old children either stay at home in the care of their family or attend kindergarten and are cared for by kindergarteners. Therefore, to reduce HFMD prevalence, preventive measures should be aimed at this age group. In addition, a high standard of personal hygiene and professional care is recommended for parents and kindergarteners of <4-years old children. Especially, children exhibiting disease symptoms should be kept away from school/kindergarten and should be allowed to recover fully at home, while being isolated.

A slight male predominance in the HFMD incidence has been reported previously[8], and we observed a similar trend in this study. The reason for this sex-based difference in disease rates is as yet unknown, and may due to the differences in boys’ habits. HFMD is transmitted through direct or indirect contact with nose and throat secretion, as well as fluid from the HFMD vesicles. Boys, in general, are more naughty, restless, and active than girls, and might have a higher possibility of being in contact with contaminated materials or surfaces; this is one possible explanation for the sex-specific difference in HFMD infection.

Considering the spatial and temporal distribution characteristics of HFMD, several researches have been conducted based on different spatial analysis models, which was helpful to understand the ecological causes of disease incidence in certain area[3, 4, 38]. Some studies found that seasonal and geographical variation could be related to climate (temperature, relative humidity, vapor pressure, duration of sunshine, etc)[39–42]. In addition, the occurrence of HFMD presents significant seasonality, i.e., temporal characteristic in Shijiazhuang City. Therefore, spatiotemporal analysis can not only help to recognize the spatial and temporal trend of HFMD, but also make prediction and guide us to formulate and implement appropriate regional public health intervention strategies to prevent and control this disease. Our research team is investigating the spatial consistence and spatiotemporal coupling between HFMD cases and pathogens based on Bayesian spatiotemporal interactive model.

Since 1997, numerous HFMD outbreaks have occurred in countries of the west pacific region where EV-A71 and CV-A16 were the most common causative EV serotypes[43–45]. Our findings are consistent with previous reports in that EV-A71 was the major aetiological agent of HFMD in Shijiazhuang City in 2010 and 2011, with the number of cases increasingly yearly, and CV-A16 became the major aetiological agent in 2009 and 2012, thus presenting a clear alternative popular pattern of these two pathogens. The slight increase in the number of HFMD cases during October–December 2011 was due to the circulation of CV-A16, which became the main HFMD epidemic-causing EV serotype in the summer of 2012, indicating a continuous increase in its prevalence. It is postulated that this cyclical pattern could be due to the accumulation of immunologically naive preschool children between epidemics until a critical threshold level is reached. Consistent with this hypothesis, a 3-year HFMD epidemic cycle (1997, 2000, 2003 and 2006), caused by EV-A71 along with the co-circulation of CV-A16, was observed in Sarawak, Malaysia[46]. Detailed characterization of the HFMD epidemics can be useful in predicting future outbreaks, and formulating control and prevention measures. However, longer surveillance periods would be needed to ascertain whether EV-A71 and CV-A16 epidemics continuously occur in 2- to 3- year cycles in the Shijiazhuang City, a northern Chinese city.

As the major causative agent among the spectrum of HFMD-causing pathogens in different areas of mainland of China, EV-A71 was believed to be the pathogen associated with most of the severe cases and almost all of the fatal cases, while CV-A16 was thought to be responsible for a sizeable proportion of the mild HFMD cases[47–49]. Similarly, in this study, EV-A71 and CV-A16 were the two most dominant pathogens in the Shijiazhuang City, while CV-A10 was the third most frequent pathogen, accounting for 4.7% of the laboratory-confirmed cases. However, the pathogen spectrum of severe HFMD cases was different; CV-A10 was the second most common pathogen, with EV-A71 being the most common EV serotype associated with serious complications. Phylogenetic analysis revealed that all EV-A71 isolates from the Shijiazhuang City HFMD cases belonged to C4a evolutionary branch, all CV-A16 belonged to B1a and B1b evolutionary branches, and all CV-A10 belonged to C genotype, all of which are common circulating genotypes in mainland of China.

Many studies have indicated that besides EV-A71, CV-A16 and CV-A10, several other EVs, such as CV-A4, CV-A6, CV-A12, CV-B3, etc., are also associated with HFMD cases and occasional outbreak events[18, 21, 24, 50, 51]. In our study, with the intensive surveillance for HFMD in Shijiazhuang City, at least 17 EV serotypes were detected to potentially contribute to HFMD cases during 2009–2012. In order to gain comprehensive knowledge of all HFMD-causing pathogens, further studies assessing the molecular characteristics of the infrequently detected pathogens are important. Enhanced enterovirus surveillances are warranted to predict the potential of these strains in causing outbreaks. The composition of the HFMD pathogen spectrum and the epidemic pattern of enteroviruses should be further detailed and comprehensively studied. Thus, our study underscores the need for enhanced surveillance and molecular detection for HFMD. Our findings also suggest that EV-A71 vaccination may be an effective public health strategy for HFMD prevention, and that vaccines against CV-A10 and CV-A16 serotypes are also urgently needed.

## Materials and methods

### Sample collection and ethics statement

This study did not involve human participants or human experimentations; the only human materials used were the throat swab and stool samples collected from suspected HFMD patients at the investigation of the Ministry of Health P. R. of China for public health purposes. Based on the latest guidelines for prevention and control of HFMD issued by the Ministry of health in China in 2009, 5 clinical samples were collected from mild HFMD patients per month per county (district) when they came to see doctors for their first time; and clinical samples were collected from all the severe and dead HFMD patients. When samples arrived in Shijiazhuang CDC, nucleic acid detection of EV-A71, CV-A16 and other enteroviruses was performed within 7 working days after receiving the samples in the laboratory using real-time RT-PCR method [52].

Written informed consents for the use of clinical samples were specifically obtained from the parents of all the children whose samples were analysed. The study was approved by the Ethics Review Committee of the Shijiazhuang CDC and was carried out in accordance with the approved guidelines.

### Epidemiological analysis and statistical analysis

The HFMD data of Shijiazhuang City during 2009–2012, including sex, age, postal address, occupation, date of illness onset, case type (mild, severe or dead) were acquired from China NNDRS. Statistical analysis was performed to describe epidemiological features including demographic characterizations, seasonal variations, and geographic distributions. The total incidence rate was defined as the total number of HFMD cases divided by the average population size during the study period. The frequencies of HFMD were summarized monthly by county (district) area, and the incidence rate of HFMD (per 100,000 populations) in each county (district) was calculated by HFMD counts divided by the corresponding population. A time series analysis was performed to evaluate the seasonal distribution of HFMD cases and to detect peaks in the number of HFMD cases. Geographic distribution was analysed using the MapInfo Pro software (version 16.0, http://www.pitneybowes.com/us/location-intelligence/geographic-information-systems/mapinfo-pro.html). The level of statistical significance were performed using the SAS software (version 9.13, SAS Institute Inc., Cary, NC, USA), and an association at the *P* < 0.05 level was considered statistically significant.

### Molecular typing of enterovirus serotypes in HFMD specimens

The collected clinical samples were processed according to standard protocols. Viral RNA was extracted from the stool and throat swab specimens using a QIAamp Viral RNA Mini Kit (Qiagen, Valencia, CA, USA), and one step reverse transcription-polymerase chain reaction (RT-PCR) was performed using the Access RT-PCR Kit (Promega, Madison, Wisconsin, USA) according to the manufacturer’s instructions. The E486 and E488 primer pairs were used to detect EV-A, and the E490 and E492 primer pairs were used to detect EV-B[53]. The PCR products were purified using a QIAquick Gel Extraction Kit (Qiagen), and the amplicons were bi-directionally sequenced using an ABI PRISM 3130 Genetic Analyzer (Applied Biosystems, Hitachi, Tokyo, Japan). Enterovirus serotypes were determined using the online Enterovirus Genotyping Tool (http://www.rivm.nl/mpf/enterovirus/typingtool)[54].

### Viral isolation and VP1 sequencing

The laboratory-confirmed HFMD specimens were inoculated into rhabdomyosarcoma (RD) or human laryngeal epidermoid carcinoma (HEp-2) cell lines. The cell lines were obtained from the WHO Global Poliovirus Specialized Laboratory, USA, and were originally purchased from the American Type Culture Collection.

Viral RNA was extracted from the culture supernatants of infected RD and HEp-2 cells that demonstrated typical cytopathic effects, using a QIAamp Viral RNA Mini Kit (Qiagen, Valencia, CA, USA). The primers used to amplify the entire viral *VP1* gene sequence have been reported previously, including primers EV-A71-VP1-S and EV-A71-VP1-S for EV-A71[26]; primers CV-A16-VP1-S and CV-A16-VP1-A for CV-A16[55] and primers CV-A10-2407Y and CV-A10-3400Z for CV-A10[18]. RT-PCR was performed using the Access RT-PCR Kit (Promega, Madison, Wisconsin, USA) according to the manufacturer’s instructions. The amplified PCR products were purified using a QIAquick Gel Extraction Kit (Qiagen), and the amplicons were bi-directionally sequenced using an ABI PRISM 3130 Genetic Analyzer (Applied Biosystems, Hitachi, Tokyo Japan).

### Phylogenetic and bioinformatics analyses

The nucleotide sequences of 13 EV-A71, 20 CV-A16 and 14 CV-A10 isolates were compared to those of representative strains of known genotypes and sub-genotypes by pairwise alignment, using the Molecular Evolutionary Genetics Analysis (MEGA) program (version 5.05, Tempe, Arizona, USA)[56]. Phylogenetic trees were constructed for the VP1 nucleotide sequences obtained from the Shijiazhuang HFMD cases and sequences downloaded from the GenBank database using the neighbour-joining method based on the Kimura 2-parameter model. The trees were based on majority-rule consensus, with 1000 bootstrap replicates shown as percentages. Bootstrap values greater than 80% were considered statistically significant for grouping.

### Nucleotide sequence accession numbers

The representative sequences of the entire VP1 region from the Shijiazhuang EV isolates (13 EV-A71, 20 CV-A16, and 14 CV-A10) study were deposited in the GenBank database under the accession numbers KY081970-KY082004, KF246659– KF246667, KF246671–KF246673.
